# Outlines of Pathology and Practice of Medicine

**Published:** 1843-04-01

**Authors:** 


### OUTLINES OF PATHOLOGY AND PRACTICE OF MEDICINE. By *William Pulteney Alison*, M.D. F.R.S.E. &c. &c. Part I. PRELIMINARY OBSERVATIONS. Part II. INFLAMMATORY AND FEBRILE DISEASES. 8vo. pp. 499. Edinburgh, 1843.

THERE is not a name in Medical Literature better known or more generally esteemed in the present day than that of Dr. *Alison*. Distinguished himself, he is one of an eminently distinguished family. Few scholars have not read and admired the beautiful disquisition of his father on Taste, so replete with fine thoughts and elegant scholarship; and his brother has already gained for himself a place among the classic authors of his age by the production of his admirable History of Europe;—a work that possesses all the fascination of a brilliant romance, and all the admonitory instructiveness of a great Moral Essay. Reared amidst so gifted a kindred, and enjoying, moreover, in early life, the inestimable advantage of having a friend and preceptor in his uncle (we believe) the late Dr. Gregory, long one of the ornaments of the northern metropolis, Dr. *Alison* started in life under the most propitious circumstances, and his career throughout has abundantly fulfilled the promise of his early years: it has been one of unbroken honour and success.

Having for several years most ably filled the chair of Physiology in the University of Edinburgh, he has recently been raised to that of the Practice of Medicine; and certainly it would be difficult to find any one so well calculated to teach the lessons of sound practical knowledge to the students of our profession.

With the exception of his distinguished townsmen, Drs. Abercrombie and Thomson, he is unquestionably the most eminent physician of the day in Scotland. There is one feature in his character that deserves especial notice; we allude to his unwearied and ever-active benevolence to the poor: he is called, we believe, emphatically the sick poor man's friend. For many years he has directed especial attention to the subject of pauperism in Scotland, and he has recently written a very able pamphlet, to prove the necessity of introducing a regular system of poor-laws among his countrymen, and in opposition to the views of his eloquent co-professor Dr. *Chalmers*. The question is likely to be soon settled, as we observe, by the public journals, that Government has just appointed a commission to inquire into and report upon the subject of having a legal provision for the poor in Scotland.

The present work bears all the impress of the author's character—calm, thoughtful, and reflective. Dr. *Alison* is not so much an original thinker, as a most wise expositor and a thoroughly honest reasoner. He has not the masculine vigour of *Elliotson*, the speculative ingenuity of *Billing*, nor the minute accuracy and clear penetration of *Brodie*. His writings appear to us to be often better adapted to those already engaged in the duties of our profession, than to the student who is endeavouring to obtain a knowledge of it. There is occasionally a degree of over-caution—bordering almost on indecision, and evidenced by the frequent use of the words

"perhaps," and "probably"—in the expression of his own opinions, which leaves the reader in the dark as to what these opinions really are. He has not the power of concentrating and arranging his ideas in that lucid manner, which leaves a picture, so to speak, on the mind of his reader: no one possesses this faculty more than Dr. Marshall Hall. But to make up for these deficiencies, our author has the inestimable superiority over too many of his cotemporaries, of never presenting any exaggerated or over-coloured statements either in the way of description or of precept. He is therefore a much safer guide than the generality of medical writers in the present day, who write more for the advancement of their personal interests, than for that of professional truth.

In one respect especially, these Outlines are calculated to do much good; they will tend to diffuse more generally the spirit of an improved Humoral Pathology in the elucidation of many diseases. *Solidism* (if we must use such a word, for want of another) is rapidly on the wane, and ere long the opposite doctrine will be more and more taught in the schools. The authority of such a man as Dr. *Alison* will contribute not a little to the acceleration of the change. Several of the extracts, which follow, will clearly show the judicious views which he holds in reference to the morbid alterations of the fluids in different diseases, and the influence which these alterations have on the progress and result of those diseases. The first, which we select, shall be on the subject of rheumatic and gouty inflammations.

After mentioning the distinctive characters of the former—its attacking certain tissues in particular, its shifting about from one part of the body to another, and its never terminating in genuine suppuration or ulceration —our author very justly remarks that, from these circumstances, "it may be suspected that there is something peculiar in the state of the blood in Rheumatic Inflammation; and it has been generally observed, that the fibrin of the blood in violent cases of Acute Rheumatism is very abundant, and its separation from the colouring matter very complete."

Gouty inflammation, at least in particular cases, has many features in common with the rheumatic; as is evident from the fact, among others, that both diseases are liable to be followed by the formation of chalk-stones around the affected joints. Still we should not consider them, we think, as degrees or modifications of the same morbid action, or as depending upon the same cause. We have no reason to believe that the proportion of the fibrine of the blood is at all changed in any case of simple uncomplicated gout; or, on the other hand, that there is necessarily any predominance of lithic acid or of its salts in the circulating or secreted fluids in pure rheumatic affections. That the two diseases are very often co-existent, and therefore that there is very frequently a two-fold morbid agency at work in the system, cannot be doubted by any one who has seen much practice; and indeed it seems to us that it is by keeping this idea steadily in view that the most rational and successful mode of treatment is suggested in a great variety of cases. Perhaps the following remark by Dr. *Alison* intimates a closer connexion between them than is strictly correct. "From the whole history," says he, "of these diseases, especially from this last fact, and from the frequent connexion of gout with gravelly deposits in the urine, it is pretty obvious that one condition necessary to the establishment of these kinds of inflammation is a morbid matter, or an excess of matter destined to excretion, elaborated in the system, and circulating in the blood."

According to our views, the *morbid matter*, here alluded to, is not the same in the two cases—in the one it is excess of fibrine, and in the other a predominance of the lithates. Before quitting this subject, let us remind our readers that, in addition to these two elements of disease, in a great number of cases of rheumatic gout or gouty rheumatism, there is a third element not unfrequently co-existing—that is, neuralgia or a painful affection of the nerves of the part. But we explained at some length our views on this question so recently,* that we shall add nothing more at present. It is satisfactory however to find that so experienced a physician as Dr. *Alison* distinctly recognises the existence of a *humoral* cause both in gout and in rheumatism.

The following important extract bears upon another branch of the subject—that form of inflammatory action which is associated with a *poisoned* or vitiated state of the blood, occasioned by the admixture with it of some hurtful matter that has been either absorbed into, or generated in, the circulation.

"There are cases in which inflammation is fatal, apparently by reason of some part of the effusions to which it gives rise, being mixed with the blood, and acting on the footing of a Poison. Thus, when inflammation is excited in a spot on the surface by the application of a specific poison, as by a wound in dissection, it is speedily attended by the formation of a similar poison, which is evidently absorbed, and excites fresh inflammation in the line of its passage into the mass of the blood; but this inflammation is attended by a peculiar *typhoid* fever, in which the heart's action is rapidly depressed, which bears no fixed proportion to the extent or intensity of the inflammation itself, and by which death may take place without visible injury of any vital organ; sometimes before the inflammation has advanced beyond its first stage, and generally long before it has gone so far as in the more usual inflammation of the same parts.

Again, in the case of inflammation of the lining membrane of a Vein, it is very often observed that the accompanying fever soon takes a similar typhoid form, often with vomiting and purging, always with a feeble or depressed state of the heart's action, as well as derangement of the nervous system. In many such cases, this typhoid form of the fever, rather than any effect which we can ascribe to the inflammation itself, appears to lead to the fatal termination; in like manner, as a similar combination of typhoid symptoms does, when occurring idiopathically, or as a part of a malignant contagious febrile disease: and this peculiarity of in-

---

* Vide the Review of M. *Bouillaud* on Rheumatism in our Number for January, 1841. We there endeavoured to point out the most important differences between rheumatic and gouty inflammations, and to explain why many cases of chronic rheumatism are so intractable under the ordinary methods of treatment. We alluded also particularly to the precursory state of the system, when the disease is *incubant*, and has not yet manifested itself. Dr. *Alison's* views seem to coincide very nearly with those which we then expressed. He observes, in his remarks on the usual causes of rheumatism, "there is no doubt some previous peculiarity in the constitution, which disposes certain individuals to be affected in this way;" and he goes on to say that several circumstances "lead us to suspect that this peculiarity consists in the constitution of the blood."

flammation of this part has been ascribed to the necessary admixture of much of the inflammatory effusion with the circulating blood, with more probability than to any other cause."

The history of many cases of large chronic abscesses, of puerperal fever, &c. will naturally occur to the mind of the medical practitioner, as he reads these most truthful observations of our author. Dr. *Alison* alludes to the not unfrequent development in cases of suppurating wounds, after small-pox, &c., of rapid secondary inflammation and effusion of pus in the internal organs (the metastatic abscesses of French writers) chiefly the lungs and liver, causing death as primary inflammation would do; but he does not touch upon the disputed question whether, in such cases, the purulent matter is always absorbed from the seat of the local disease, and whether there is invariably an accompanying phlebitis—as alleged by several writers of the present day. In the last Number of this Journal, pages 222-8, the reader will find some interesting remarks on this subject from the recently-published memoirs of MM. *Tessier* and *Blandin* of Paris.

As a good specimen of the preceptive character of our author's writings in matters of practice, we select the following passage on the occasional effects of losses of blood, whether artificial or not, upon the system. He has been inculcating the necessity of vigorous depletion in cases of active inflammation, and of watching the effects of the treatment on the pulse: then he adds the important caution.

" It must be allowed that there are many cases in which the system is powerfully affected by loss of blood, in which the repetition of the remedy is dangerous, if not immediately, at least in its ultimate result in the disease; and in which, nevertheless, there is a fallacious degree of fulness and even strength of the pulse, and a combination of symptoms which to those unaccustomed to observe them might seem to denote determination to the head, perhaps inflammation of the brain, and to demand farther loss of blood.

" Of the possibility of this fallacious fulness and even sharpness of the pulse (generally a somewhat tremulous and easily compressed, but nevertheless sharp pulse, according to the notion formerly explained as being annexed to this last term), some of the experiments of Dr. Parry on animals killed by repeated bleedings, and in which the pulse was ' full and bounding' almost to the moment of death, afford unequivocal proof. And many practical observations by Rush, Armstrong, Marshall Hall, Travers, and others, illustrate this ' reaction after the loss of blood;' which may perhaps be most correctly described as a modification of the inflammatory fever, produced in a great measure by the loss of blood, and persisting after the local inflammation has subsided, or passed into a state no longer demanding evacuation. This peculiar febrile state is marked by the frequent, full, vibrating, or sharp, but easily compressed pulse, with heat of skin, generally, however, not persistent if the bedclothes are removed from the part felt,—generally with sense of palpitation and of throbbing in the head, and tinnitus aurium; sometimes impatience of light and sound;—the symptoms aggravated, and vertigo produced by the erect posture; the face and lips pale, and all muscular motion difficult and generally tremulous. This state occurs chiefly in females of irritable constitution; and is best relieved by alternation of laxatives and opiates, often with the cautious use of wine, ammonia, or other stimuli. Where it co-exists, as occasionally happens, with still urgent symptoms of local inflammation, it presents a case of much difficulty, but in which, although blood may often still be taken locally, general bloodletting is certainly dangerous." 231.

The obstetrical physician has more frequent opportunities than any other of appreciating the accuracy and practical value of these remarks, as the series of symptoms so well described by our author is very often observed after great uterine hæmorrhages. In the treatment of such cases, Dr. A. omits to mention the necessity of absolute rest and quietude both of mind and body—perhaps the most important precept of all. It is truly astonishing to notice the difference in the progress and result of similar cases in different patients, according to the character of their temper and character, whether these be cheerfully calm and resigned, or impatient and desponding. The importance of proper religious feeling under such circumstances is immense.

Dr. *Alison* is evidently not favourable to the Continental practice of administering the tartrate of antimony in large doses, as a contra-stimulant, in internal inflammations: this practice has never been fairly established in this country. He rightly prefers the English method of giving it in quarter or half-grain doses every two hours or so, to induce and keep up a state of constant nausea. Thus used, it is, as he observes, the most powerful auxiliary to blood-letting in the early stages of inflammations within the chest, especially in that of the substance of the lungs; it is moreover a very powerful remedy in those cases of affection of the brain occurring in fever, in which there is a high delirium and an approach to inflammation, though without nausea.

In closing his directions on the treatment of inflammation in general, the author makes some admirable remarks on the necessity of using stimulants freely in certain cases, where either the duration of the disease itself, or some other circumstance, has induced much prostration and tendency to a fatal sinking of the powers of life. In the advanced stage of enteritis for example, when the pain ceases, the pulse gives way, and the surface becomes cold and clammy, the patient, as Dr. *Abercrombie* has convincingly shewn, may sometimes be saved by the cautious but continued use of stimuli. In bronchitis, also, if the breathing becomes very short and hurried, and the pulse tremulous, and when the mucous and subcrepitous râles are heard on both sides of the chest, these alarming symptoms will be occasionally relieved by like treatment—certainly not by any other. In such cases, there is usually an accumulation of mucus in the air-passages, and unless the system has strength sufficient to expectorate this freely, life will inevitably sink.

"Again," adds our author, "although there be no such immediately alarming symptoms, if bronchitis has produced general effusion into the bronchiæ (as in many advanced cases of asthma and of hooping-cough); if a portion of lung has been consolidated; if an extensive and probably partly puriform effusion from decided inflammation has taken place in the cavity of one side of the chest or abdomen; if an abscess has formed in the liver; if a portion of the mucous membrane of the intestines has been thickened by effused lymph, and then passed into ulceration; if a bone has become carious, a cartilage has ulcerated, or even a capsular ligament of a joint been much thickened by inflammatory deposits; if the cornea has been affected, first with pustules and then with ulcers, from the strumous form of ophthalmia,—whatever influence local remedies may or may not have on such lesions, it is certain that a long process of absorption, of ulceration, of healing by granulations, &c. in these different cases, must be gone through; and that a certain degree of strength of habit is necessary, that these processes may go on favourably." 250.

In the chapter on Nephritis, there are some interesting remarks on the disease of albuminuria, and, as the profession may naturally wish to know the sentiments of so calm a thinker as Dr. *Alison*, we gladly select the following extract, wherein he gives a succinct sketch of the conclusions to which he has come from his own experience, and the study of the best authorities on the subject.

"The most important facts ascertained as to the subsequent progress of cases in which this morbid state of the Kidneys exists, are the following.

"1. That when the urine, although albuminous and of low specific gravity, is in quantity greater than natural (so that the usual amount of solid matter may pass off from the kidneys in a given time,) the general health may be tolerably good for a considerable number of years; although there is a liability to very various diseases, making a very cautious regimen necessary.

"2. That many such persons are found to be simultaneously affected with other diseases, chiefly of the liver or of the heart, which are originally of the same character, and which of course increase their liability to disease.

"3. That such persons are very liable, particularly on exposure to cold, to attacks of Dropsy.

"4. That they are also peculiarly liable (with or without such dropsical attacks) to sudden inflammation, especially of the chest.

"5. That they are liable to organic diseases of the brain.

"6. That many of them are subject to sickness and vomiting, independently of liver disease, and especially to diarrhœa; those who are subject to the diarrhœa being perhaps the least liable to the dropsical affections.

"7. That when the quantity of urine becomes less (as very generally happens in the progress of such cases,) and its specific gravity and the quantity of solid matter and even of albumen in it diminish at the same time, we must expect, not only that these complications will become more frequent and intractable, but that they will be attended with more or less of the characteristic effects of the presence of Urea in the nervous system, particularly drowsiness, spasms, indistinct vision, &c. and ultimately delirium and fatal stupor." 331.

In the consideration of the various morbid phenomena which are apt to be induced by, or at least to be associated with, this peculiar state of the urinary secretion, the physician will, as a matter of course, never lose sight of the important fact, that the constitution of the blood is usually found to have undergone great alterations from the standard of health; its normal proportion of albumen, and subsequently of its colouring matter also, being decidedly diminished, while at the same time it contains, at least in some cases, a certain quantity of urea.

No subject has engaged more, or perhaps so much, of our author's attention than that of Fevers; and, as might be expected, the whole of the chapter, which he devotes to their consideration, is full of sound and most useful observation. We need scarcely say, that he avoids all *exclusivism*, either in theory or practice, in reference to this class of diseases, and that he entirely rejects the notion of so many writers of the French school, that they are merely the results or effects of any local lesion, whether in the intestines or elsewhere. He repeatedly speaks of a morbid agent at work within the system, that is quite independent of any local inflammatory mischief, either in the head, chest, or abdomen, and which exerts a depressing influence on the vital energies of the body, as manifested by that train of symptoms to which the appellation of *typhoid* has been applied.

This morbid agent he views in the light of a *poison*, that has been absorbed into or generated within the system, and of which one of the most constant effects, is to induce an alteration in the constitution of the blood: but whether its primary operation is on the nervous or on the vascular system, he does not presume to determine.

"In a great majority of the cases in which we see typhoid fever, we are sure that some peculiar matter, generally absorbed from without, must be contained in the blood; as in the case of fever from malaria, from contagion (whether of simple fever or the eruptive fevers) from inflamed veins, from animal poisons introduced by wounds, or from suppression of the natural excretion at the kidneys. That this peculiar matter, or the blood altered by it, should act like a ferment, assimilating much of the circulating fluid to itself, in the former case equally as in the latter, is quite in accordance with what has been observed, when purulent matter has begun to form in the blood." 439.

It is unnecessary to say that so accurate an observer as our author fully recognises the frequency of local inflammation in the course of many fevers: but then he does not fail to shew, at the same time, that the inflammatory action, when present—for it is not necessarily and essentially so—is always more or less modified, both in its characters during life and in its effects as found after death, from the idiopathic and uncomplicated inflammation of the same organ.

Hence we derive the important practical deduction, that the same mode of treatment that is proper in the one case, may be quite unsuitable in the other.*

Dr. A. recognises three forms of simple or idiopathic fever;—1. The congestive, as in the bad cases of plague, yellow fever, cynanche maligna, &c. 2. The inflammatory (synochus); and, 3, the typhoid. Of this latter, he describes three varieties, which may certainly be distinguished in some cases, but which generally are blended together, or graduate into one another. The author's own words will convey his meaning best.

"*a.* When the most obvious and urgent of the typhoid symptoms are those of mere debility of the vital actions,—soft compressible pulse, dry foul tongue and lips, deficient or easily depressed heat of the surface, and extreme muscular debility, shewn in the voice and attitude as well as in the muscular movements, —the name of 'Fievre Adynamique,' is given by recent French authors, and the term Low Fever is the most appropriate in our language.

"*b.* When the most obvious and urgent of the typhoid symptoms are those indicating derangement of the functions of the Nervous System,—pervigilium, restlessness, tremors or spasms, deafness, contracted pupil, and other affections of the external senses, delirium, especially of the more active kind, and this after-

---

* According to the experience of Dr. *Alison*, serous effusion within the cranium is, probably, the most frequent morbid phenomenon in the continued fever of Scotland. With respect to the follicular enteritis, so much insisted upon in the writings of the continental physicians, he says, that it is found in a much smaller proportion of fatal cases in Edinburgh, than either in London or Paris.

wards subsiding into stupor—the case has been styled 'Fievre Ataxique' by the French, and is generally called Nervous or Brain Fever in this country. This form of fever is most remarkably seen in persons in whom the nervous system has been previously and habitually excited, either by voluntary muscular or mental exertion, or strong and lasting emotion,—or by the inordinate use of stimuli, such as alcohol.

"*c.* When the most obvious of the typhoid symptoms are those denoting a dissolved state of the blood, petechiæ, passive hæmorrhages, gangrene from slight irritations, &c. the case has still among many the name of Putrid Fever." 398.

He does not agree with those authors (and we observe, Dr. *Copland* is one,) who attempt to draw a distinction between typhus and typhoid fever, grounding the distinction chiefly on the presence, in the former, of a peculiar exanthematous eruption, which the learned author of the Dictionary describes to be "as characteristic of it (typhus) as the eruptions of measles or of scarlatina."

We think Dr. A. is quite correct in his opinion that—

"The differences observed are only varieties depending on constitution, and on the agency of other causes affecting the constitution, besides the exciting cause of the disease." 447.

We have no intention of entering at present upon the wide subject of the treatment of typhoid fever, and merely wish to caution the young practitioner against ever pushing the use of depletory and evacuating remedies for the relief of the local phlogistic affections in fever, so far as in the idiopathic inflammations of the same organs. Too much attention cannot be paid to the type or general character not only of each epidemic, but also of each individual case, in the management of all fevers. Of late years, medical men have often been far too timid in the use of stimulants, allowing their minds to be continually haunted with the idea of inflammation in some part or another, even when the circulation was becoming languid and the energies of the nervous system were giving way. Not so our experienced author; for he expressly says:—

"Whenever we observe the circulation to become feeble,—or even (in Epidemics where we know that much debility is to be expected) before it has become feeble,—we use the stimulants,—chiefly wine,—in bad cases, spirits, ammonia, or æther,—in small but frequent and gradually-increasing doses,—both during and after the time when the remedies are applied to the local affections, in the hope of maintaining the requisite strength of the circulation, until the time when the spontaneous favourable change is to be expected: and although many of the cases, in which the circulation becomes very feeble, are fatal, we see many recover, both from the simpler and more complex forms, to whom the stimuli are given in such quantity as cannot be supposed to be inert; and in whom the pulse becomes less frequent and firmer under their use." 462.

With this we close our notice of the present volume, and shall not fail to make our readers acquainted with the contents of the second, whenever it is published.

